# Prevention of irradiation-induced salivary hypofunction by rapamycin in swine parotid glands

**DOI:** 10.18632/oncotarget.7941

**Published:** 2016-03-06

**Authors:** Zhao Zhu, Baoxing Pang, Ramiro Iglesias-Bartolome, Xiaoshan Wu, Lei Hu, Chunmei Zhang, Jinsong Wang, J Silvio Gutkind, Songlin Wang

**Affiliations:** ^1^ Molecular Laboratory for Gene Therapy and Tooth Regeneration, Beijing Key Laboratory of Tooth Regeneration and Function Reconstruction, Capital Medical University School of Stomatology, Beijing 100050, China; ^2^ Oral and Pharyngeal Cancer Branch, National Institute of Dental and Craniofacial Research, National Institutes of Health, Bethesda, MD 20852, USA; ^3^ UC San Diego, Department of Pharmacology and Moores Cancer Center, La Jolla, CA 92093, USA

**Keywords:** radiotherapy, salivary hypofunction, miniature pig, rapamycin, mTOR

## Abstract

Radiotherapy is commonly used in patients with oral cavity and pharyngeal cancers, usually resulting in irreversible salivary hypofunction. Currently management of radiation damage to salivary glands still remains a great challenge. Recent studies show that activation of mammalian target of rapamycin (mTOR) occurs in salivary gland lesions, making it possible to apply mTOR inhibitor for treatment. Our results indicate inhibition of mTOR by rapamycin significantly alleviated irradiation-induced salivary hypofunction by restoring 46% salivary flow rate and protecting histological structures in swine. Furthermore, rapamycin protected human submandibular gland cell line (HSG) from irradiation-induced cell depletion and loss of cell proliferation capacity. These findings lay the foundation for a new clinical application of rapamycin to prevent irradiation-induced salivary hypofunction.

## INTRODUCTION

It is estimated that the incidence rate of lip, oral cavity and pharynx cancers was 48.1 per 10,000 in China in 2015 [1]. Radiotherapy is commonly used to treat the majority of patients with oral cavity and pharyngeal cancers, but often results in radiation exposure of the normal salivary gland, inducing irreversible irradiation-induced hypofunction. Because of the loss of salivary flow, patients suffer a series of side effects such as dental caries, mucositis and dysphagia, along with a significant increase in per-patient care costs [2–5]. The new radiotherapy technology image guide radiation therapy (IGRT) and various radio-protectants, such as Amifostine or Tempol, could significantly alleviate irradiation-induced salivary gland injury [6–9], however, these therapies are not widely available and remain palliative in essence.

Our previous studies have shown that miniature pig (minipig) parotid glands are similar with human glands in anatomic and physiologic characteristics, and this has provided a valuable large animal model to investigate irradiation-induced salivary hypofunction [10, 11]. Partial function restoration has been achieved using adenoviral-mediated transfer of the human aquaporin-1 cDNA (AdhAQP1) to irradiated minipig parotid glands, and adeno-associated viral -mediated transfer of the human aquaporin-1 cDNA (AAV2AQP1) could extend the efficiency [12, 13]. In addition, treatment with an adenoviral vector encoding fibroblast growth factor-2 (AdFGF2) resulted in the protection of parotid microvascular endothelial cells from IR damage and significantly limited the decline of parotid salivary flow [14]. However, viral vector-mediated gene transfer is a relatively complicated process, and needs further investigations such as long-term efficiency, safety and other factors.

The mammalian target of rapamycin (mTOR), a highly conserved serine/threonine protein kinase, is as a key regulator of cell growth, protein synthesis, metabolism homeostasis and cancer progression [15–17]. Activation of the mTOR pathway occurs after salivary gland injury by physical and chemical insults [18, 19]. It has been shown that activation of mTOR can lead to exhaustion of particular cell populations, including tissue-repopulating stem cells, due to differentiation or senescence [20–22]. Based on this information, we investigated whether the selective mTOR inhibitor rapamycin could aid in the prevention of irradiation-induced hypofunction of minipig parotid gland. We found that rapamycin administration significantly prevented irradiation-induced salivary hypofunction in minipigs. Furthermore, mTOR inhibition protected HSG cells from cell depletion and loss of proliferative capacity following irradiation. This protection was accompanied by a decrease in DNA damage and upregulation of mitochondrial superoxide dismutase (MnSOD), likely resulting in reduced radiation induced-reactive oxygen species (ROS).

## RESULTS

### Prevention of irradiation-induced hyposalivation by rapamycin

**Figure 1:**
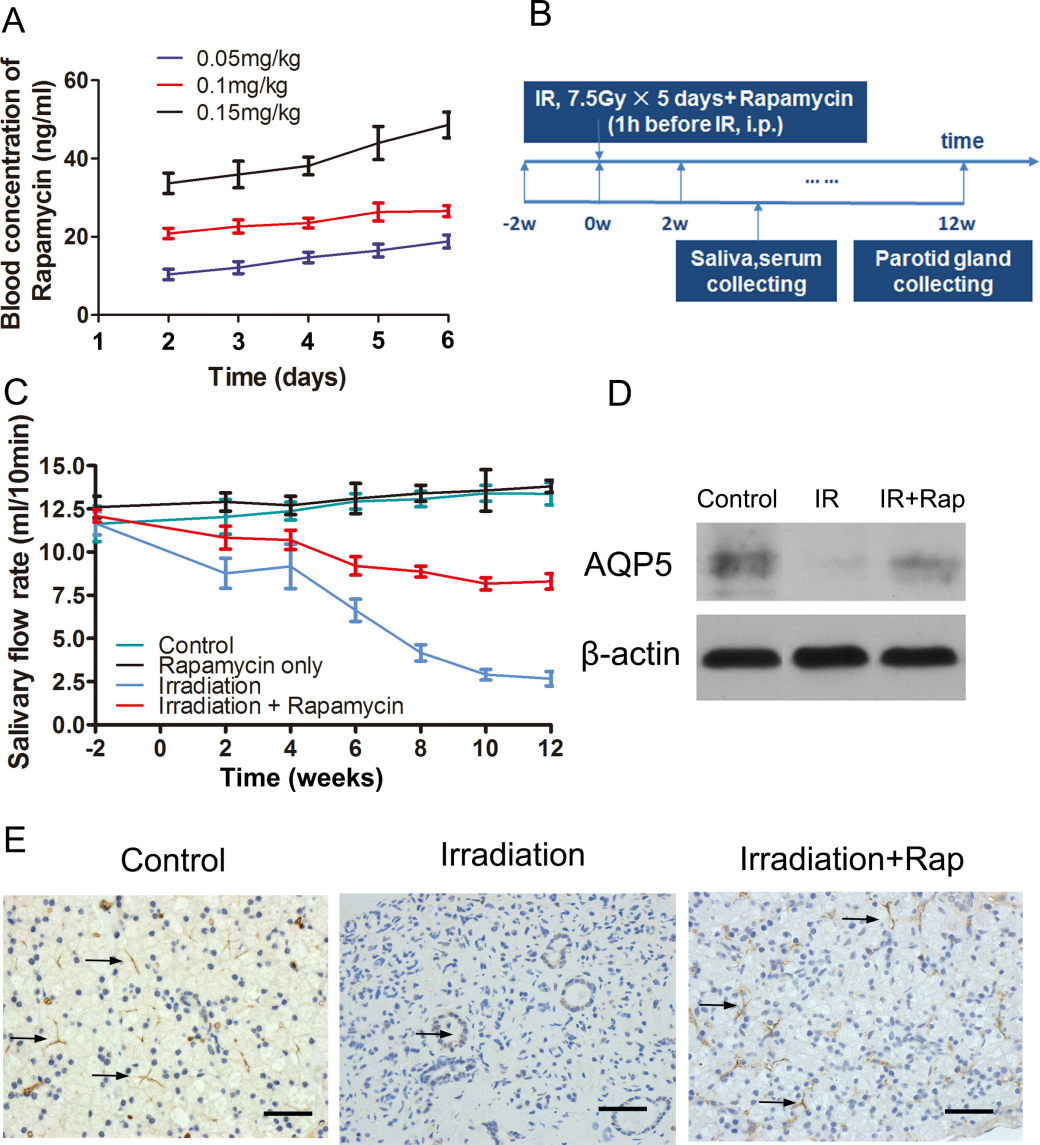
Prevention of irradiation-induced hyposalivation by rapamycin (**A**) blood concentration of rapamycin in minipigs during the treatment process to determine the ideal injection dose. The dose of 0.1 mg/kg could keep the ideal blood trough level of rapamycin at 20 ng/ml in the 5 days. (**B**) scheme of the procedures guided for this study. Saliva and serum were collected in every 2 weeks, and then minipigs were sacrificed at the 12th week after irradiation. (**C**) salivary flow rate comparison among control, rapamycin only, irradiation and irradiation + rapamycin groups. Parotid saliva was collected from targeted parotid gland. The Rapamycin dose was same in 2 experiment groups. Data shown were the mean values ± s.d. of parotid saliva flow rate (ml/10 min, *n* = 3 per group). (**D**) and (**E**) comparison of AQP5 expression among control, irradiation and irradiation + rapamycin groups with Western Blot assay (normalized to β-actin, *P* < 0.001) and immunohistochemistry assay. AQP5 labeling was seen in apical membranes of the acinar and duct cell region (black arrows pointing to the AQP5 positive region, bars, 50 μm).

Initially, we determined the optimal rapamycin dose to inject animals in order to achieve therapeutic levels of drug in blood. In humans, 20 ng/ml of rapamycin is a well-tolerated dose and can lead to a persistent blockade of mTOR with limited toxicity [23, 24]. To determine the dose of rapamycin to achieve this rapamycin blood levels, nine minipigs were dived into 3 groups (*n* = 3 per group), which received 0.05, 0.1 or 0.15 mg/kg injection dose per day for 5 continuous days separately. Blood samples were collected before each injection from the 2nd to the 5th day and blood samples were taken at the same daytime at the 6th day. We found that the dose of 0.1 mg/kg was able to maintain a blood level close to 20 ng/ml throughout the 5 day treatment (Figure 1A).

Next, we explored whether rapamycin could prevent irradiation-induced salivary gland hypofunction following radiation treatment. Twelve minipigs were used in this study (4 groups, *n* = 3 per group). Parotid gland areas of minipigs where irradiated with a fractionated irradiation schedule of 7.5 Gray (Gy) /day for 5 days. Before each irradiation, minipigs were injected with vehicle or rapamycin (0.1 mg/kg/day) intraperitoneally. Saliva of parotid gland and blood samples were collected fortnightly after irradiation (Figure 1B). There was no significant difference of salivary flow rate between control group and rapamycin only group (mean ± s.d.: 13.37 ± 0.49 ml/10 min vs 13.80 ± 0.27 ml/10 min, *t* = 1.018, *P* = 0.37). Both of these two groups did not accept irradiation, which indicated that rapamycin treatment in the normal condition didn't affect salivary output. Twelve weeks after irradiation when the salivary flow rate was stable, the salivary flow rate in irradiation group was sharply reduced by ∼77% (Figure 1C; 11.67 ± 0.51 ml/10 min, before IR vs 2.67 ± 0.31 ml/10 min, 12 weeks after IR, *P* < 0.0001). However, the saliva output in irradiation + rapamycin group (Figure 1C; 12.1 ± 0.27 ml/10 min, before IR vs 8.3 ± 0.33 ml/10 min, 12 weeks after IR, *P* < 0.001) was significantly elevated by ∼46% compared with the irradiation group. Additionally, there was a marked increase in expression of acinar markers Aquaporin 5 (AQP5) with rapamycin treatment, as shown by western blot and immunohistochemistry (Figure 1D, 1E). All together, these results indicate that rapamycin treatment remarkably protects the secretion function of parotid gland in minipigs after fractionated irradiation.

### Histological assessments of minipigs parotid gland

**Figure 2:**
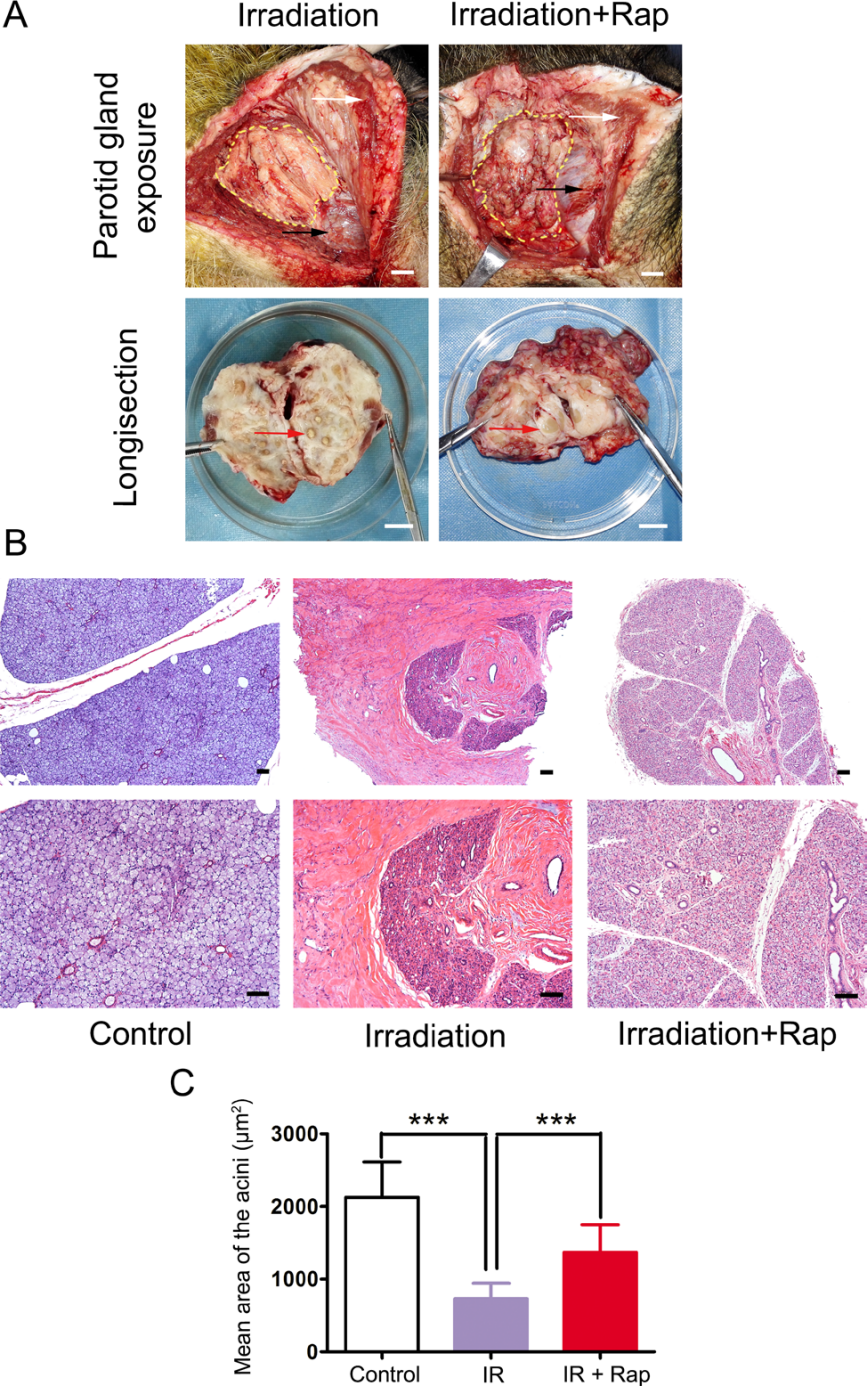
Histological assessments of parotid glands 12 weeks after fractionated irradiation (**A**) comparison of parotid gland gross pathology between irradiation and irradiation + rapamycin groups. The yellow dashed-line regions showed parotid gland exposure after the flap raised. The anatomical marks: platysma (white arrows) and the trailing edge of masseter on mandibular angle (black arrows). The parotid glands of two groups was dissected and compared in the 10 cm-diameter dish. Red arrows showed the acinus in the longisection view (bars: 1 cm). (**B**) H & E staining of parotid glands of three groups. The structure of glandular lobule and ductal system were preserved in irradiation + rapamycin group, compared to the obvious fibrosis in irradiation group (bars, 100 μm). (**C**) comparison of mean area of the acini with H & E staining morphometric analysis. Twenty acini per group were randomly selected and calculated the acini size under 200× magnification with Image-Pro Plus 6.0. The acini size in irradiation group significantly decreased in contrast to control group (***P < 0.0001). The Irradiation + Rapamycin group was significant increase in contrast to irradiation group (***P < 0.0001). Data was expressed as mean ± s.d.

Twelve weeks after irradiation, minipigs were sacrificed and the parotid gland tissues were removed to analyze the effect of rapamycin treatment. At a macroscopic view analysis, the border line of parotid gland was not clearly defined in irradiation group. In addition, the texture was hard and severe fibrosis was found in most of the gland region. Only few atrophic acini were left in the longitudinal section view. Surprisingly, the fibrotic degeneration of parotid gland tissue in irradiation + rapamycin group was significantly alleviated by rapamycin treatment, in which the texture and gland macro-structure were similar to those of a normal, non-irradiated parotid gland (Figure 2A).

Histological analysis of haematoxylin and eosin (H & E) stained sections showed intensive congeneric serous secretory acinus in control group, as has been previously reported [11]. On the contrary, the glands in irradiation group were characterized by significant reduction of acinar number, acinar atrophy and duct luminal dilation. The structure of glandular lobule disappeared, being replaced by numerous adipose and fibrosis tissue. However, the glands in irradiation + rapamycin group maintained the features of acinar cells, although irradiation-associated reduction in acinar size and numbers was evident. In addition, we noted that there were no apparent changes in the ductal system compared with the control group (Figure 2B).

Morphometric analysis of H & E staining showed that the acini size in irradiation group (mean area ± s.d.: 733.05 ± 166.56 μm^2^) significantly decreased (*P* < 0.0001) compared to control group (2126.66 ± 316.46 μm^2^). The acini size was remarkably rescued in irradiation + rapamycin group (1372.36 ± 252.88 μm^2^) (*P* < 0.0001) (Figure 2C).

### Clinical chemistry assessments

Hematological examination. There were two transient hematology parameter value differences after fractionated irradiation between with and without rapamycin treatment. The number of white blood cells decreased in irradiation group compared with irradiation + rapamycin group at week 4 post-irradiation, and returned to normal range at week 12. Moreover, the number of platelets increased at 4 weeks post-irradiation only in irradiation group. However, there were no significant differences between the values examined before IR and 12 weeks after irradiation. Rapamycin at 0.1 mg/kg/day did not have any significant side effects in blood routine examination (data not shown).

In salivary chemical values shown in Table 1, there were some significant alterations, for instance, decline of amylase, Ca^2+^ and Na^+^; and ascent of K^+^ in irradiation group which in accordance with our previous study [11, 12]. In the contrary, rapamycin preferably maintained the balance of composition of saliva obtained from control, non-irradiated parotid glands.

**Table 1:** The representative chemistry values of saliva obtained from parotid glands

	Non-Irradiation	Post-Irradiation (12 weeks)	
	**Control**	**Irradiation**	**Irradiation + Rapamycin**
**Amylase (IU/L)**	1153 ± 161	688 ± 152*	1202 ± 133^ns^
**Ca2+ (mmol/L)**	3.0 ± 0.2	1.7 ± 0.1***	1.8 ± 0.1***
**Cl− (mmol/L)**	16.2 ± 1.1	17.1 ± 1.4^ns^	16.8 ± 1.7^ns^
**K+ (mmol/L)**	24.2 ± 2.8	28.1 ± 2.1^ns^	25.4 ± 1.8^ns^
**Na+ (mmol/L)**	12.9 ± 1.6	8.33 ± 1.5*	10.1 ± 1.8^ns^

Data shown were the mean ± s.d. of saliva chemistry values (*n* = 3 per group). Data analyzed by the two irradiation groups compared with the control group respectively and used a one-way analysis of variance, followed by the Turkey test.

* *P* < 0.05

*** *P* < 0.001

ns non-significant.

### mTOR changes after irradiation

**Figure 3:**
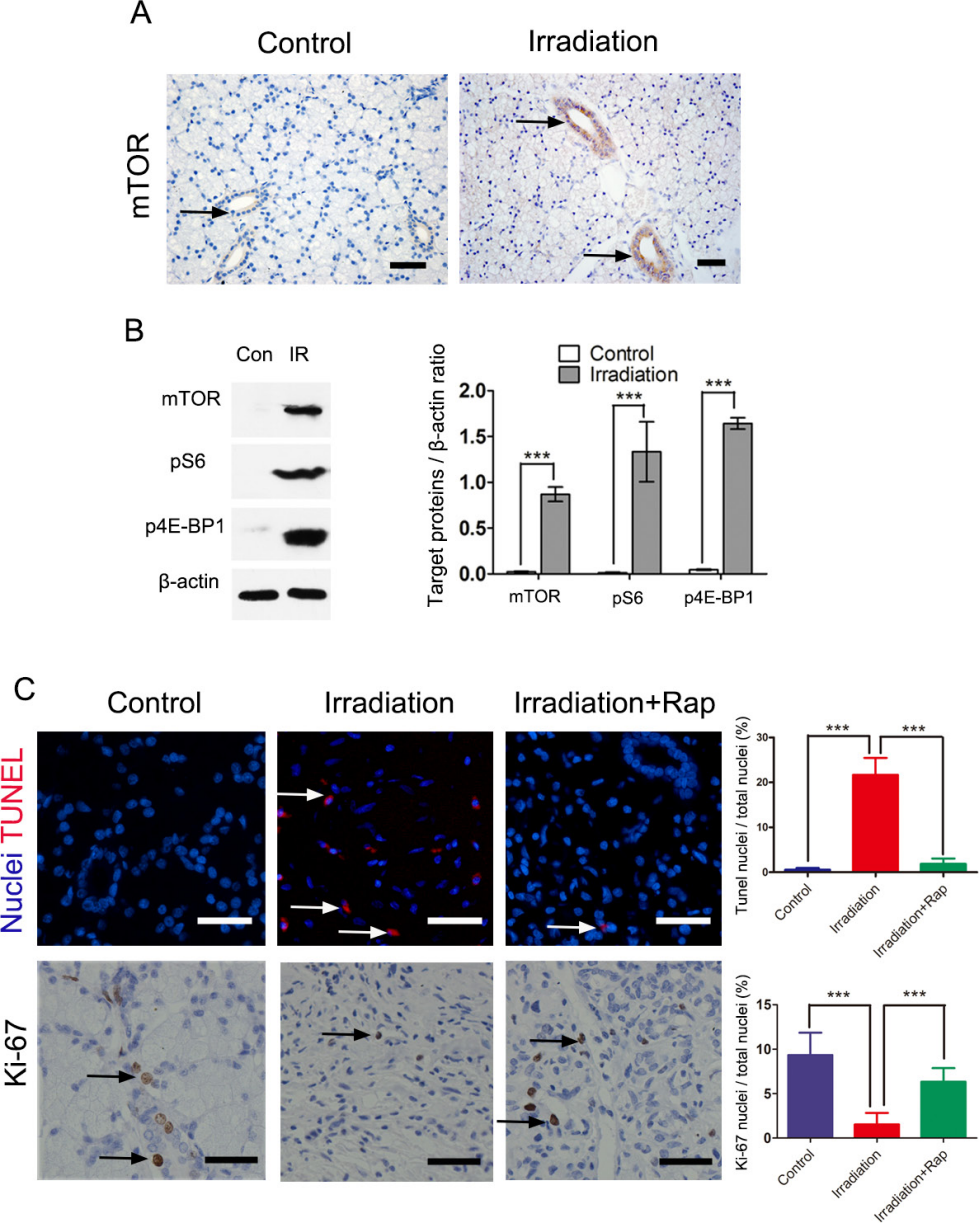
Irradiation induced activation of mTOR pathway immediately after 5 days fractionated irradiation treatment (**A**) immunohistochemical staining for expression of mTOR in control and irradiated parotid gland. The expression of mTOR after irradiation was more significantly expressed in ductal cells region than that of control group (black arrows showing the positive mTOR labeling, bars, 50 μm). (**B**) comparison of expression of mTOR, pS6 and p4E-BP1 between control and irradiation groups with western blot assay. The graph represented the ratio of mTOR, pS6 and p4E-BP1 to total β-actin by densitometry (***P < 0.0001). (**C**) TUNEL and Ki-67 assays of parotid gland 12 weeks after irradiation, showing significantly reduced apoptotic cells (White arrows showing the apoptosis nuclei, bars, 50 μm) and preserved tissue proliferative function in irradiation + rapamycin group (Black arrows showing the Ki-67 positive nuclei bars, 50 μm). The proportion of positive cells was detected by fluorescence microscopy and quantified using ImagePro. Data was expressed as mean ± s.d.

We found that the mTOR pathway was activated in salivary gland following fractionated irradiation treatment. In normal conditions, mTOR pathway is usually switched off in salivary glands. Previous studies have reported activation of mTOR following salivary gland injury, including duct ligation [18], irradiation [19] and salivary gland tumors [25]. In the present study, we found the levels of mTOR expressed in ductal cells cytoplasm of parotid gland of minipigs increased immediately after fractionated irradiation (Figure 3A). In addition, western bolt analysis provided a strong evidence of mTOR activation. Both phosphorylated S6K and 4E-BP1 [26, 27], two vital downstream targets of activated mTORC1, were over-expressed following radiation treatment (Figure 3B). Twelve weeks after irradiation, TUNEL analysis showed that the apoptotic cells reduced significantly compared to the irradiation group (*P* < 0.0001), and Ki-67 assay indicated that treatment with rapamycin preserved tissue proliferative function (*P* < 0.0001) (Figure 3C).

### mTOR inhibition by rapamycin restrains cell proliferation of HSG cells

**Figure 4:**
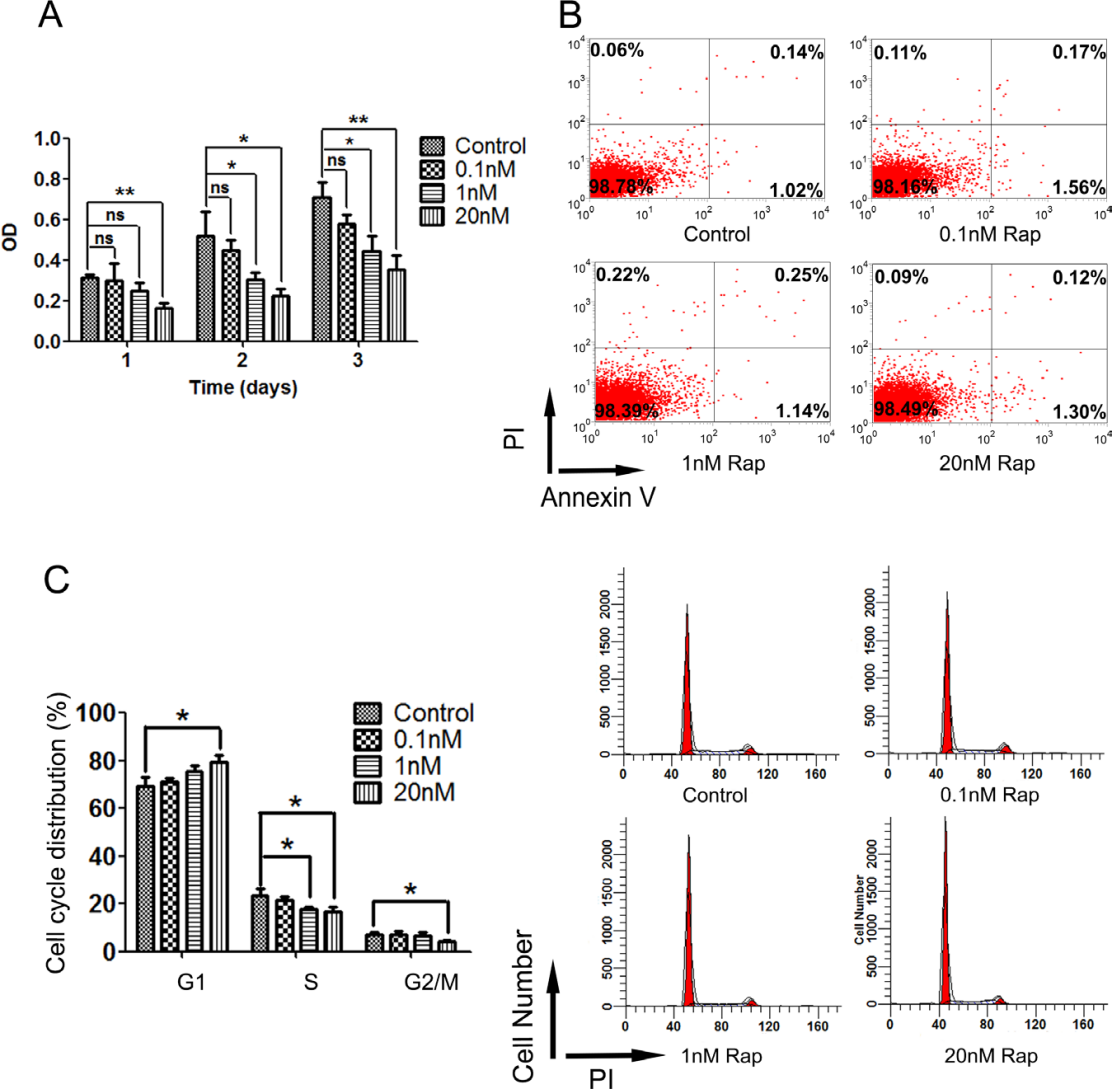
Cell proliferation, apoptosis and cell cycle assays of HSG cells after 3 days treatment of rapamycin Cells were plated and treated with different concentration or without of rapamycin for 72 h. (**A**) labeling of cell proliferation by CCK-8 after 72 h rapamycin treatment, then detected the optical density (OD) in 450 nm by microplate reader. Rapamycin treatment restrained the cell proliferation of HSG cells (*P < 0.01; **P < 0.001). (**B**) apoptosis assay of HSG cells 24 h after 72 h rapamycin treatment by AnnexinV. Rapamycin treatment didn't affect cell apoptosis of HSG cells. (**C**) analysis of cell cycle 24 h after 72 h rapamycin treatment by flow cytometry. Rapamycin treatment prolonged G0–G1 phase of HSG cells (*P < 0.05).

Based on the results above, we next investigated the protective mechanisms of rapamycin in HSG cells. For this, cell proliferation, apoptosis and cell cycle were examined to investigate the effect of rapamycin on HSG cells. Treatment with rapamycin decreased the cell proliferation rate (*P* < 0.01) (Figure 4A). We did not find any significant differences in the number of apoptotic cells detected by AnnexinV compared to cells without rapamycin treated as control group (Figure 4B). Finally, we found that rapamycin restrained HSG cells in G0-G1 phase (Figure 4C). These findings suggest that rapamycin may lead HSGs to quiescence.

### mTOR inhibition protected HSG cells from losing proliferation capacity and decreased DNA damage response induced by reactive oxygen species following irradiation

**Figure 5:**
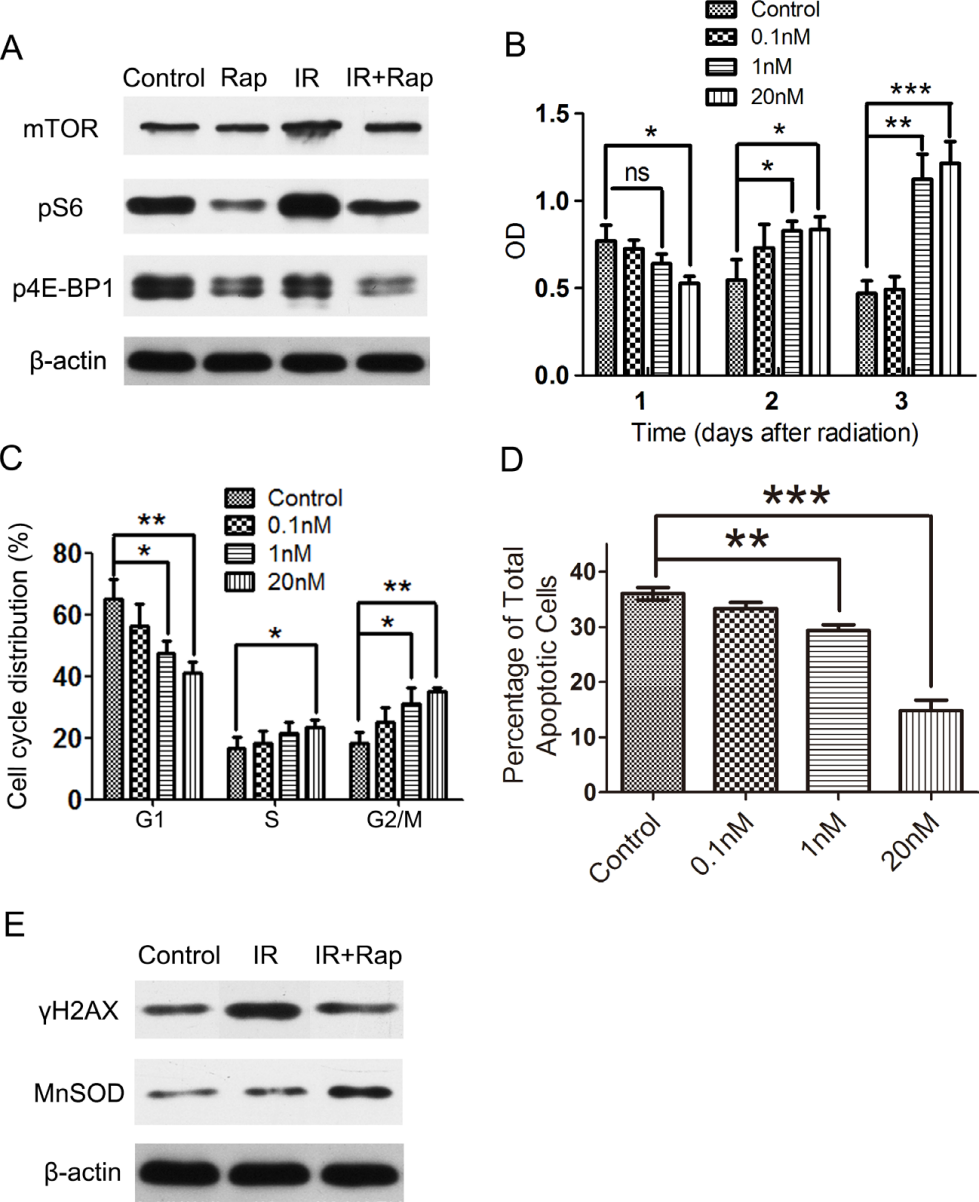
Rapamycin protected the proliferation capacity and decreased ROS-induced DNA damage response in irradiated HSG cells following irradiation Cells were plated in 6 wells plates and treated with different concentration or without of rapamycin for 72 h, then irradiated with 8 Grays (Gy), and maintained in vehicle or rapamycin medium for 24 h, afterwards all cells were changed into normal medium. (**A**) western blot analysis of mTOR pathway expressions in control (non-irradiated and no rapamycin treatment, Control), rapamycin treated (non-irradiated, Rap), irradiation (IR) and irradiation + rapamycin (IR + Rap) HSG cells 24 h after irradiation. Rapamycin incompletely inhibited mTOR pathway activation of HSG cells following irradiation. (**B**) labeling of cell proliferation by CCK-8 after irradiation. After 8 Gy irradiation, when cells changed into normal medium, treatment with rapamycin significant increased cell proliferation compared with control group (irradiated HSG cells without rapamycin treatment) (*P < 0.05; **P < 0.01; ***P < 0.001). (**C**) analysis of cell cycle 24 h after irradiation by flow cytometry. The percentage of rapamycin treated irradiated HSGs in G2 stage was higher than control (irradiated HSG cells without rapamycin treatment) (*P < 0.05; **P < 0.01). (**D**) apoptosis assay of HSG cells 24 h after irradiation by AnnexinV. Rapamycin treated irradiated HSGs significantly decreased cell apoptosis. (**E**) western blot analysis of γH2AX and MnSOD expression in HSG cells after irradiation. Rapamycin treatment cells (IR + Rap) decreased the expression of DNA damage marker γH2AX compared with irradiated HSG cells (IR), meanwhile, increased the expression of ROS scavenger MnSOD (Control represents non-irradiated and no rapamycin treated HSG cells).

By analyzing the effect of radiation on mTOR activity, we found that mTOR was activated in HSG cells and expressed more significantly after irradiation. Rapamycin almost completely blocked this radiation-induced mTOR activation, as analyzed by western blot of mTOR, phosphorylated S6K and 4E-BP1 in non-irradiated and irradiated HSGs (Figure 5A).

Next, we investigated whether rapamycin could protect HSGs from irradiation injury. HSG cells were plated and treated with or without different concentrations of rapamycin for 72 h, then received a single dose of 8 Gy irradiation, and were then maintained in vehicle or rapamycin treated medium for 24 h. After that, all the cells were changed into normal medium and different assays were performed. Surprisingly, rapamycin significantly improved the proliferation capacity of HSG cells after irradiation (Figure 5B). In addition, the proportion of rapamycin treated HSG cells in G2 phase was significantly higher than that of control (vehicle-treated cells) 48 h after irradiation (Figure 5C). Interestingly, the total apoptotic rate of rapamycin treated HSGs was significant lower than that of control (vehicle-treated cells) (Figure 5D). These findings indicate that mTOR inhibition by rapamycin could retain the proliferative capacity of HSG cells following irradiation, and decrease irradiation-induced cell death.

We have previously shown that increased expression of mitochondrial-localized superoxide dismutase (MnSOD) induced by rapamycin may enhance irradiation resistance by means of scavenging ROS in normal tissues [28, 29], but not in cancer cells [30]. We find that the protein level of MnSOD is increased in rapamycin treated cells after irradiation. Furthermore, the reduction of ROS level also can alleviate the DNA damage response. Indeed, we found reduced levels of the DNA damage marker γH2AX after rapamycin treatment (Figure 5E). These findings suggest that rapamycin protect the proliferative capacity of HSG cells after irradiation, probably by decreasing a radiation-induced DNA damage response.

## DISCUSSION

Radiotherapy for oral cavity and pharyngeal cancers often generates salivary gland hypofunction. Efforts to reduce the therapeutic radiation complications have focus on both radiotherapy technology and radio-protectors. New radiotherapy technology gives a maximum accuracy and alleviates normal tissue injury. However, still two-thirds of oral cavity and pharyngeal cancer patients whose salivary glands are included in or proximate the radiation area will suffer salivary hypofunction [31]. Meanwhile, amifostine, is not recommended in head and neck malignancies due to its uncertain tumor protection [32, 33].

Our previous studies have proven the antitumoral activity of rapamycin in many experimental systems [5, 34, 35] and is currently being tested in multiple clinical trials. Additionally, it almost completely prevents the appearance of irradiation-induced mucositis in mice [30]. In the present study, we show that rapamycin can preserve the integrity of the glandular lobule following irradiation, which is in contrast with the severe fibrosis and acinar atrophy observed in the irradiation group. We also detect an early activation of mTOR in ductal system after irradiation. This ductal systems may be the residence of salivary gland stem/progenitor cells [36–38] and activation of mTOR untimely lead to stem cell differentiation or senescence. Of direct clinical relevance, these beneficial effects were achieved keeping the level of rapamycin in blood during the fractionated irradiation process at its therapeutic levels (20 ng/ml), which is effective and safe in accordance with clinical use of prevention of kidney transplant rejection [39]. Based on our current data and previous publications, we hypothesize that inhibition of mTOR activation may protect the loss of salivary stem/progenitor cells, thereby promoting the recovery of salivary gland function after irradiation.

In order to show that this protection could be translated into a human salivary gland system we use HSG cells in our study [40]. Our results indicate that mTOR inhibition could also protect HSG cells from irradiation-induced cell depletion and loss of proliferative capacity. It is known that cells are relatively sensitive to radiation during mitosis process and a previous study showed that irradiation can lead to a decreased in cell proliferation of HSGs, and an arrest in G2/M phase [41]. In this study, rapamycin delayed G0-1 and S phase of HSGs before irradiation [42], which contributed to a temporary growth arrest, probably allowing successful DNA repair after irradiation [43–45]. Meanwhile, we find rapamycin treatment following radiation could reduce accumulation of DNA damage response marker H2AX. The DNA damage response is a major mediator of cell senescence [46–48]. Rapamycin also increased expression of MnSOD, a scavenger of mitochondrial ROS, which are activated following radiation treatment. This is in agreement with previous results in irradiated human primary oral keratinocytes [30]. ROS accumulation often causes multiple lesions of DNA double helix, ultimate leading to DNA double-strand breaks [49, 50]. It is possible that rapamycin prevent stem/progenitor cells in ductal system from undergoing senescence following irradiation by decreasing ROS-induced DNA damage response.

In summary, using a large animal model of fractionated irradiation-induced salivary hypofunction and an *in vitro* model of human salivary gland, we show that mTOR inhibition by rapamycin treatment results in salivary gland radioprotection which hints at a new clinical application. Since rapamycin is already being used for treatment in a wide range of diseases, we could envision its potential clinical application for salivary gland protection.

## MATERIALS AND METHODS

### Animals

Healthy littermate male minipigs, ∼ 8 months old, weighing 25∼35 kg, were obtained from the Institute of Animal Science of the Chinese Agriculture University (Beijing, China). Animals were fed under conventional conditions, as reported previously [12]. This study was reviewed and approved by the Animal Care and Use Committee of Capital Medical University.

### Rapamycin treatment

Rapamycin (LC Laboratories) was dissolved in a stock solution at 20 mg/ml in 100% ethanol and stored −80°C. For injection, rapamycin (0.1 mg/kg) was diluted in 30 ml 1×PBS, containing 5.2% (v/v) PEG (polyethylene glycol) 400 (Sigma) and 5.2% (v/v) Tween80 (Sigma) for one dose per day. We used Architect Sirolimus Reagent Kit (Abbott Laboratories) to measure the level of rapamycin in blood in swine. This kit is commonly used as an aid in the management of renal transplant patients receiving rapamycin therapy.

### Fractionated irradiation of parotid glands

We performed axial computerized tomographic scans to determine the irradiation (IR) plan using a three-dimensional treatment planning system (Pinnacle3, version 7.6; ADAC Inc., Concord, CA, USA). Calculations showed that more than 95% of the irradiation dose covered the whole target volume of the parotid gland. The reference point for all dose calculations was the center-targeted parotid gland. Animals were injected with rapamycin (0.1 mg/kg) intraperitoneally 1 hour before being irradiated with 7.5 Gy/day for 5 days. We used image guide radiation therapy (IGRT) technology in the IR group. Before irradiation, the real-time image of the parotid gland area by iViewGT should coincide with the plans which have been determined, and then the imaging workflow was integrated into the system. Thereafter, the animals were irradiated with 6 mV of photon energy at 3.2Gy/min by Elekta Synergy accelerator (Elekta Synergy^®^; Elekta AB, Stockholm, Sweden).

### Collection of saliva and blood

Parotid saliva was collected and salivary flow rates determined, as described previously, using a modified Carlson–Crittenden cup [14] on anesthetized animals following an intramuscular injection of pilocarpine (0.1 mg/kg). In this study, we used a mechanical vacuum device (Shanghai Zhang Dong Medical, Shanghai, China), connected with the Carlson–Crittenden cup. Parotid saliva was collected from each parotid gland of all animals for ∼10 min on the time points indicated in the Results section. Meanwhile, blood was obtained from the precaval vein.

### Histologic and immunohistochemical analyses

Twelve weeks after irradiation the animals were sacrificed, the parotid glands were removed, cut into multiple pieces (∼5 × 5 × 5 mm^3^), and fixed in 4% paraformaldehyde, after being dehydrated with gradient ethanol, the samples were embedded in paraffin, and then sectioned at 4 μm thickness. The sections were either stained with hematoxylin and eosin for the evidence of pathological changes or processed immunohistochemical staining for the evidence of AQP5 (1:200 Santa Cruz), mTOR (1:150 Cell Signaling Technology) or Ki-67(1:100 Abcam) expression.

### Western blot analyses

Parotid glands and HSG cells were homogenized in RIPA buffer with 5 mM sodium orthovanadate (Fisher Scientific), protease inhibitor cocktail (Sigma-Aldrich) and 100 mM PMSF (Fisher Scientific). The samples were then boiled for 10 minutes and sonicated until homogenous. 100 mg of each protein sample was added to 12% polyacrylamide gels and transferred to 0.45 mm Immobilon-P membranes (Millipore Billerica, MA). Membranes were blocked using non-fat dry milk or 5% BSA then immunoblotted with one of the following antibodies: AQP5 (1:1000, Santa Cruz), total mTOR (1:1000, Cell Signaling Technology), pS6 (1:1000, Cell Signaling Technology), p4E-BP1 (1:1000, Cell Signaling Technology), γH2AX (1:1500, Santa Cruz), and MnSOD (1:500, Santa Cruz).

### Clinical laboratory analyses with blood and saliva samples

The collected saliva and blood samples were analyzed by standard clinical procedures. Hematology analyses included the number of white blood cells, red blood cells, platelets, hematocrit, concentration of hemoglobin, mean corpuscular volume, % lymphocytes, % monocytes and % granulocytes. Parotid saliva samples were analyzed for total protein, albumin, amylase, Na^+^, K^+^, Cl^−^, Ca^2+^ and PO_4_^3−^.

### Cell culture, proliferation, apoptosis and cell cycle

HSG cells (obtained from Dr. M. Sato, Tokushima University, Japan) were cultured in minimum essential medium α (MEM α) (Gibco-Invitrogen) supplemented with 5% fetal bovine serum (FBS) and antibiotics, at 37°C in the presence of 5% CO_2_.

Cell proliferation was evaluated by Cell Counting Kit-8 (CCK-8) (Dojindo Laboratories, Japan). Briefly, HSG cells were plated in 96 wells (100 μl/well) and treated with rapamycin (20 nM, 1 nM, 0.1 nM) or vehicle for 3 days. If received irradiation, cells were cultured in medium with rapamycin or vehicle for 24 h, and then all the cells were changed into normal medium. Afterwards, 10 μl of the CCK-8 solution was added into each target well. After Incubation for 2 hours, the absorbance of wells was measured at 450 nm using a microplate reader.

Cell apoptosis was detected using Annexin V-FITC Apoptosis Detection Kit (BD Bioscience). Cells were plated in 6 wells and treated as described above. Cells were digested by EDTA-free 0.25% trypsin, centrifuged at 1100 rpm for 5 minutes, removed the supernatant and added 200 μL Annexin V binding buffer. Added 2 μL Annexin V-FITC into every tube, incubated for 15 minutes, and then added 5 μL PI. FACS analysis was performed in a FACS Calibur flow cytometer (BD Bioscience). Tissue apoptosis was detected using the Cell-Light™ EdUTP Apollo^®^567 TUNEL *in Situ* Detection Kit (Ribobio).

Cell cycle assay was performed by FACS Calibur flow cytometer (BD Bioscience). Briefly, cells were treated as the above mentioned, harvested and fixed in 70% ethanol overnight at 4°C, centrifuged, and removed the supernatant. Then the cells were washed with PBS and incubated with RNase A, DNase, and protease-free solutions (Fermentas) for 30 min to remove RNA. Last, the cells were stained with PI and flow cytometry was used to detect the cell-cycle phase.

### Statistical analysis

Data shown are the mean values ± s.d. All statistical calculations were performed using one-way ANOVA followed by Tukey multiple-comparison test. Statistical analysis and graphical generation of data were done with the GraphPad Prism software.
